# Impacts of Cyclosporin A on clinical pregnancy outcomes of patients with a history of unexplained transfer failure: a retrospective cohort study

**DOI:** 10.1186/s12958-021-00728-x

**Published:** 2021-03-16

**Authors:** Danni Qu, Xiangming Tian, Ling Ding, Yuan Li, Wenhui Zhou

**Affiliations:** Medical Center for Human Reproduction, Beijing Chao-Yang Hospital, Capital Medical University, 100020, Beijing, People’s Republic of China

**Keywords:** Transfer failure, Cyclosporine A, Clinical pregnancy, Obstetric outcomes, Perinatal outcomes

## Abstract

**Background:**

A rapid development in assisted reproductive technology (ART) has led to a surge in its popularity among target couples. However, elucidation on the molecular mechanism and effective solutions for a common problem posed by ART, namely transfer failure, is still lacking. The new therapeutic potential of cyclosporin A (CsA), a typical immunosuppressant widely used in the treatment of rejection after organ transplantation, in recurrent pregnancy loss (RPL) patients may inspire some novel transfer failure therapies in the future. To further explore the clinical effects of CsA, this study investigated whether its application can improve clinical pregnancy outcomes in patients with a history of unexplained transfer failure in frozen-thawed embryo transfer (FET) cycles.

**Methods:**

Data from a retrospective cohort investigation (178 frozen-thawed embryo transfer cycles in 178 patients) were analysed using binary logistic regression to explore the relationship between CsA treatment and clinical pregnancy outcomes; the odds ratios (ORs) and 95 % confidence intervals (CIs) were calculated as a measure of relevancy. Implantation rate was the main outcome measure.

**Results:**

There was no difference in the fine adjusted OR (95 % CI) of the implantation rate [1.251 (0.739–2.120)], clinical pregnancy rate [1.634 (0.772–3.458)], chemical pregnancy rate [1.402 (0.285–6.909)], take-home baby rate [0.872 (0.423–1.798)], multiple births rate [0.840 (0.197–3.590)], preterm birth [1.668 (0.377–7.373)], abnormal birth weight [1.834 (0.533–6.307)] or sex ratio [0.956 (0.339–2.698)] between the CsA-treated group and control group. No birth defects were observed in the present study.

**Conclusions:**

Although CsA does not affect infant characteristics, it has no beneficial effects on the clinical pregnancy outcomes in patients with a history of unexplained transfer failure in FET cycles.

.

## Background

While assisted reproductive technology (ART) has greatly improved outcomes in human infertility, it has also presented some new challenges, such as transfer failure, which is common. Although it has been indicated that various factors, including some healthy lifestyle habits, are involved in the success of embryo implantation, it has been gradually acknowledged that a perfect synchrony between a healthy blastocyst and the receptive endometrium is the main factor responsible for the success [1–8]. However, due to a lack of knowledge on the detailed molecular mechanism underlying the crosstalk between a blastocyst and endometrium, effective treatment methods for transfer failure in clinical practice are still scarce.

Cyclosporine A (CsA) induces immune tolerance via a broad biological impact on various immune cells. It is a typical immunosuppressant widely used in the treatment of rejection after organ transplantation and some autoimmune diseases [9–19]. Further, owing to its modulatory effect on apoptosis and proliferation, CsA is also used as a potential therapeutic measure in other medical conditions such as neuronal damage, hepatic failure, myocardial infarction and congenital muscular dystrophy [20–24]. Overall, the effects of CsA on the human body are far more complicated and comprehensive than those known to researchers.

Successful implantation and normal pregnancy depend on a perfect collaboration between foetus-derived trophoblasts and various mother-derived cells. This collaboration establishes a unique immune microenvironment that supports foetal development until delivery. Desynchronisations between the embryo and endometrium, such as those caused by immune disturbance (excessive activation of immune competent cells at the maternal-foetal interface) and/or dysfunctions of trophoblasts are closely involved in several pregnancy-related complications, including recurrent implantation failure, miscarriage, preeclampsia, foetal growth restriction, and so forth [1, 25–28]. Our previous *in vitro* studies confirmed a favourable modulatory effect of CsA on human first-trimester trophoblast cell function. Another animal study suggested a beneficial effect of CsA on maternal-foetal immune tolerance and pregnancy outcome improvement in abortion-prone matings [29–31]. These results indicate a new therapeutic potential of CsA as a trophoblast function enhancer and a simultaneous immune microenvironment regulator [29–31], which may shed light on the treatment of transfer failure. However, whether CsA can improve embryo implantation rate in patients with transfer failure remains unclear.

Thus, the present study retrospectively analysed the clinical pregnancy outcomes in patients with a history of unexplained transfer failure categorized either in the conventional luteal support therapy group or the CsA luteal support therapy group. We hope this study can better assess the clinical application value of CsA and provide guidance, as well as new ideas, for transfer failure treatment.

## Methods

### Patients

We enrolled 178 women with at least one prior transfer failure who underwent frozen embryo transfer (FET) cycles with (n = 58) or without (n = 120) CsA administration from February 2017 to December 2019 in the Medical Center for Human Reproduction, Beijing Chao-Yang Hospital, Beijing, China. A retrospective cohort study was then performed to analyse the clinical pregnancy outcomes of patients in FET cycles. This retrospective study was approved by the Human Research Ethics Committee of Beijing Chao-Yang Hospital; all patients agreed to provide their therapy information.

The inclusion criteria were: patients who received conventional *in vitro* fertilization (IVF) or introcytoplasmic sperm injection (ICSI) and also accepted ≥ 1 transfer cycle with at least 1 high-quality embryo transferred per cycle but have not succeeded in pregnancy yet. The exclusion criteria include: (1) uterine factors: uterine malformations, post myomectomy of submucous myomas, intrauterine adhesions and other factors affecting the endometrium; (2) embryo factors: poor embryo quality (scored less than grade 3); (3) patients with rescue ICSI or testicular and epididymal sperm extraction; (4) pelvic endometriosis, endometrial cyst of the ovary or adenomyosis, etc.; (5) other systemic factors, including antiphospholipid syndrome (APS), pre-thrombolic state (PTS), autoimmune diseases (AID).

### Frozen‐thawing embryo transfer procedure

Endometrial preparation methods included natural and artificial cycles according to our previously described protocols [32]. Progesterone (Zhejiang Xianju Pharmaceutical. Co. LTD, Hangzhou, China) of 80 mg was injected intramuscularly to transform endometrium when endometrium thickness was more than 7 millimeters (mm). Progesterone support was kept to the day of serum human chorionic gonadotropin (HCG) examination.

The warming process was performed according to the instruction of the manufacture (KITAZATO). Briefly, the cryopreservation carriers were directly inserted into the pre-warmed warming thawing solution (37 °C) for 1 min. Then the warmed embryos were suspended in dilution solution, washing solution 1 and washing solution 2 for 3, 5 and 1 min. at room temperature, respectively. Embryos were generally transferred on day 3 or day 5 after being incubated for 2 h.

Embryos were graded according to our previous publication, using Istanbul consensus and the Gardner scoring systems [32–35]. In order to eliminate the effects of embryo quality on pregnancy outcome, only embryos transferred in the 8-cell stage or at least one embryo transferred in the 8-cell stage on day 3 of the cleavage stage and embryos graded over 3BB on days 5–6 in the blastocyst stage were recruited in the present study.

### CsA addition

Embryo implantation involves a series of complicated biological processes and there are no specific guidelines regarding the application of CsA in transfer failure treatment. Therefore, the attending physician made the decision whether to use CsA based on the inclusion and exclusion criteria stated before. In addition, the patient couple had the right to make the ultimate decision whether or not to receive CsA treatment after they had thoroughly learned the possible benefits and drawbacks of the therapy.

Conventional luteal phase was supported with intramuscular progesterone injections of 80 mg / day and kept to the day of blood pregnancy test. In the CsA group, besides conventional tocolytic therapy, the oral administration of 50 mg CsA (Hang Zhou Zhong Mei Hua Dong Pharmaceutical Co., Hang Zhou, China) in the morning, noon, and evening was kept from the day before embryo transfer (ET) to the day of blood pregnancy test. Moreover, Patients with positive HCG (blood value ≥ 25 IU/mL) kept receiving CsA treatment to the day of ultrasound confirmation of clinical pregnancy.

### Outcome measurements

Serum ß-HCG was measured on the 12-14th day after transfer. A positive result was followed by an ultrasound scan of the uterus at gestational weeks 7–8. All of the infants were evaluated for gestational age, gender, birth weight, and defects at delivery. Pregnancy outcomes were collected from each couple by two professional follow-up personnels [32–34].

Pregnancy outcomes were recorded, including: implantation rate (IR) = the number of implanted embryos/that of transferred embryos, clinical pregnancy rate (PR) = the number of patients with clinical pregnancy/that of transferred patients, chemical pregnancy rate = the number of patients with chemical pregnancy/that of transferred patients and take-home baby rate = the number of cycles with live birth/that of transfer cycles. Positive HCG is a value of serum HCG ≥ 25 IU/mL on the HCG examination day. Biochemical pregnancy means positive HCG but an absence of gestational sac by ultrasound detection. Clinical pregnancy means the presence of intrauterine gestational sac by ultrasound at 7–8 gestation weeks [32–34].

### Statistics analysis

The data were reported as the mean ± standard deviation (SD), median (interquartile range) (IQR) or % (number/total). Statistical analyses were carried out using SPSS statistics 20.

The baseline characteristics of patients including age, body mass index (BMI), basal follicular stimulating hormone (FSH) value, causes of infertility, the number of ET cycles, endometrium thickness, endometrium morphology, the number of total embryos cryopreserved and prior embryos transferred, the number of current cycle embryos transferred, development stage of ET embryos were compared with descriptive analysis.

Binary logistic regression was preformed to explore the effects of CsA treatment on clinical pregnancy outcomes, including IR, PR, take-home baby rate, singleton /multiple births rate, gestation weeks at birth, birth weight and sex ratio. Analyses were adjusted for potential confounders, such as age, BMI, number of ET cycles, number of ET embryos, endometrium thickness and the stage of embryos. The ORs and 95 % CIs were calculated as a measure of relevancy.

## Results

### Baseline characteristics of the patients

The general clinical information of patients in FET cycles included in the CsA (58 cycles) and control (120 cycles) groups is summarised in Table 1. No statistical differences in the patients’ age (years) at oocyte pick-up [32.0 (29.0-34.3) vs. 31.5 (29.0–35.0), *P* = 0.698], patients’ age during ET [33.5 (30.0-35.3) vs. 32.0 (30.0–36.0), *P* = 0.777], body mass index (BMI,kg/m^2^) [22.5 ± 3.3 vs. 22.8 ± 3.5, *P* = 0.596], basal follicular stimulating hormone (FSH) value (IU/mL) [5.9 (4.3–7.4) vs. 6.0 (5.0–8.0), *P* = 0.225], number of total embryos cryopreserved [6 (4–8) vs. 5 (3–6), *P* = 0.138] and causes of infertility (*P* > 0.05) were found between the two groups. However, the number of ET cycles [2.0 (2.0–3.0) vs. 2.0 (2.0–2.0), *p* = 0.027] and the number of total prior embryos transferred [2.0 (2.0–2.0) vs. 2.0 (1.0–2.0), *p* = 0.006] in the CsA group were higher than those in the control group. In addition, we presented the conditions of the embryos and the endometrium of patients in FET cycles, including the number [2.0 (2.0–3.0) vs. 2.0 (2.0–2.0), *P* = 0.230] and development stage of the embryos (*P* > 0.05) selected for ET, endometrium thickness (mm) [9.0 (8.0-10.3) vs. 9.0 (8.0-10.5), *P* = 0.632] and endometrium morphology (*P* > 0.05). No significant differences were observed in these parameters between the two groups.

**Table 1:** Baseline characteristics of patients

Characteristic	CsA (*N* = 58)	Con (*N* = 120)	*P* value
Patients’ age at OPU (y)	32.0 (29.0-34.3)	31.5 (29.0–35.0)	0.698
Patients’ age during ET (y)	33.5 (30.0-35.3)	32.0 (30.0–36.0)	0.777
BMI (kg/m^2^)	22.5 ± 3.3	22.8 ± 3.5	0.596
Basal FSH (IU/mL)	5.9 (4.3–7.4)	6.0 (5.0–8.0)	0.225
No. of ET cycles	2.0 (2.0–3.0)*	2.0 (2.0–2.0)	0.027
No. of total embryos cryopreserved	6 (4–8)	5 (3–6)	0.138
No. of current cycle embryos transferred	2.0 (2.0–3.0)	2.0 (2.0–2.0)	0.230
No. of total prior embryos transferred	2.0 (2.0–2.0)*	2.0 (1.0–2.0)	0.006
Endometrium thickness(mm)	9.0 (8.0-10.3)	9.0 (8.0-10.5)	0.632
Causes of Infertility			> 0.05
Female (%)	65.5 (38)	69.2 (83)	
Diminished ovarian reserve (%)	13.1 (5)	14.5 (12)	
Ovulation disease (%)	18.4 (7)	19.3 (16)	
Tubal disease (%)	63.2 (24)	60.2 (50)	
Unexplained (%)	5.3 (2)	6.0 (5)	
Male (%)	1.7 (1)	5.8 (7)	
Female and Male (%)	27.6 (16)	22.5 (27)	
Others (%)	5.2 (3)	2.5 (3)	
Primary (%)	48.3 (28)	55.0 (66)	
Secondary (%)	51.7 (30)	45.0 (54)	
Development stage of ET embryos			> 0.05
Day3 (%)	46.6 (27)	50.0 (60)	
Day5 (%)	15.5 (9)	14.2 (17)	
Day6 (%)	37.9 (22)	35.8 (43)	
Endometrium morphology			> 0.05
A (%)	58.6 (34)	58.3 (70)	
A- (%)	20.7 (12)	31.7 (38)	
Others (%)	20.7 (12)	10.0 (12)	

Values are shown as mean ± SD, median (IQR) or n (%). *P < 0.05 (*P* value*: Kruskal Wallis Rank Test for continuous variables) compared with the control group. *CsA* cyclosporin A; *OPU* oocyte pick-up; *ET* embryo transfer; *BMI* body mass index; *FSH* follicular stimulation hormone

### Effects of CsA on clinical outcomes

**Fig. 1:**
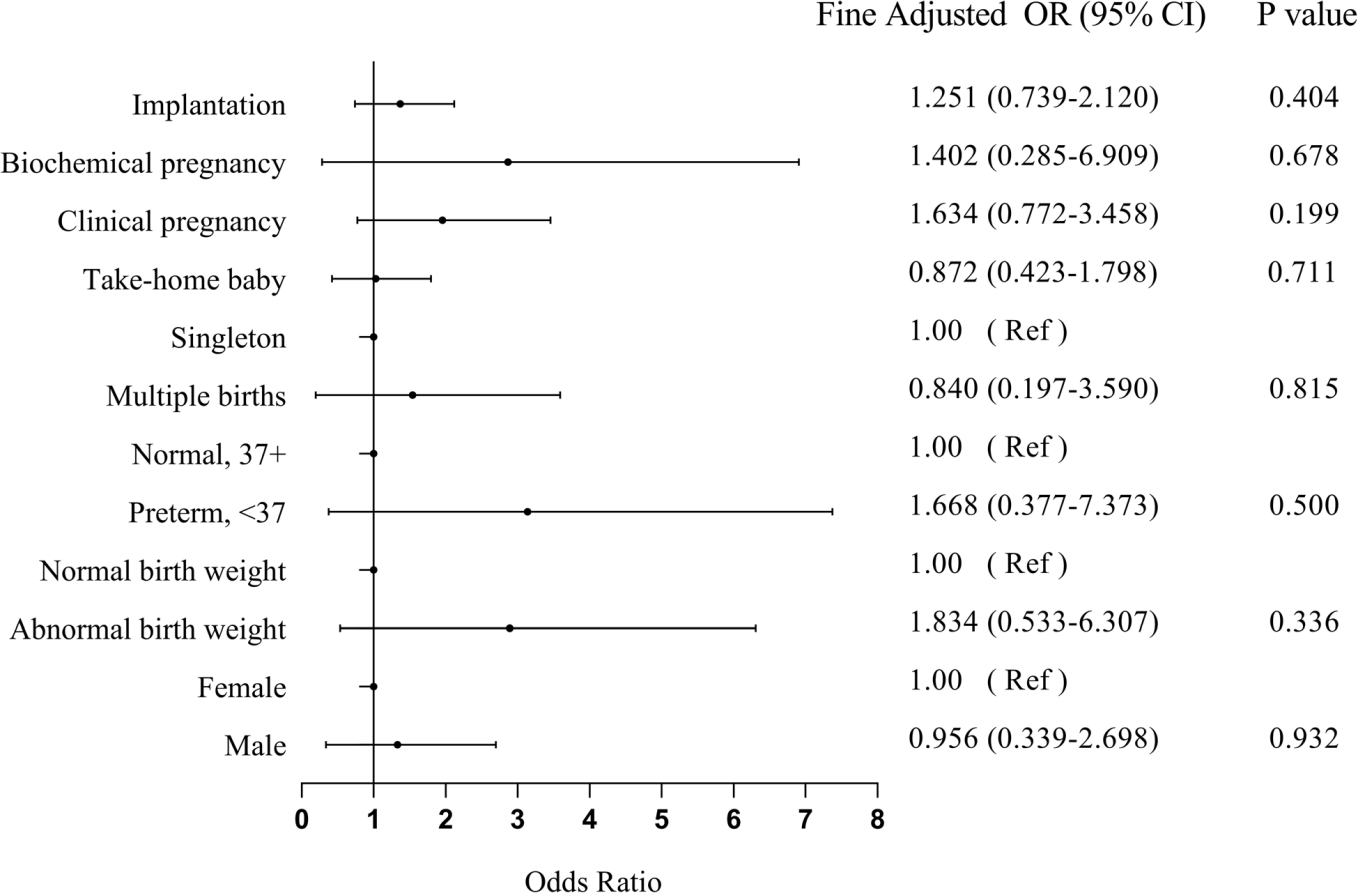
Clinical outcomes following CsA treatment vs. conventional treatment

The clinical outcomes of the patients are included in Table 2; Fig. 1. A total of 50 out of 107 transferred embryos implanted in the CsA group versus 83 out of 212 in the control group. CsA showed no effect on the implantation rate [46.7 % vs. 39.2 %, *P* = 0.404] after adjusting for maternal age, BMI, number of ET cycles, number of embryos transferred, endometrium thickness, and embryo stage [fine adjusted OR 1.251,95 % CI 0.739–2.120]. The clinical pregnancy rate was higher in the CsA group, but there was no significant difference between the two groups [63.8 % vs. 47.5 %, *P* = 0.199, fine adjusted OR 1.634, 95 % CI 0.772–3.458]. A similar result was also observed in the biochemical pregnancy rate [5.2 % vs. 5.8 %, *P* = 0.678, fine adjusted OR 1.402, 95 % CI 0.285–6.909].

**Table 2:** Clinical outcomes of patients following CsA vs. conventional treatment

Outcome	CsA	Con	Unadjusted OR (95 % CI)	Rough Adjusted OR (95 % CI)	Fine Adjusted OR (95 % CI)
Implantation rate^a^ (%)	46.7 (50/107)	39.2 (83/212)	1.363 (0.853–2.180)	1.134(0.678–1.898)	1.251 (0.739–2.120)
Biochemical pregnancy^a^ (%)	5.2 (3/58)	5.8 (7/120)	0.881 (0.219–3.536)	1.176 (0.264–5.232)	1.402 (0.285–6.909)
Clinical pregnancy^a^ (%)	63.8 (37/58)	47.5 (57/120)	2.194 (1.140–4.224)	1.574 (0.762–3.249)	1.634 (0.772–3.458)
Take-home baby rate^a^ (%)	44.8 (26/58)	38.3 (46/120)	1.307 (0.693–2.466)	0.848 (0.416–1.728)	0.872 (0.423–1.798)
Singleton rate^a^ (%)	80.8 (21/26)	71.7 (33/46)	1.00	1.00	1.00
Multiple births rate^a^ (%)	19.2 (5/26)	28.3 (13/46)	0.604 (0.188–1.943)	0.795 (0.218–2.901)	0.840 (0.197–3.590)
Gestation at birth (wk)^a^ (%)					
Normal, 37+	73.1 (19/26)	80.4 (37/46)	1.00	1.00	1.00
Preterm, < 37	19.2 (5/26)	19.6 (9/46)	1.082 (0.318–3.684)	1.355 (0.357–5.144)	1.668 (0.377–7.373)
Birth weight (kg)^a^ (%)					
Normal weight, 2.5-4.0	72.4 (21/29)	74.6 (44/59)	1.00	1.00	1.00
Abnormal weight	27.6 (8/29)	25.4 (15/59)	1.117 (0.410–3.047)	1.195 (0.408–3.499)	1.834 (0.533–6.307)
Very low birth weight, < 1.5	6.9 (2/29)	0 (0/59)			
Low birth weight, < 2.5	13.8 (4/29)	18.6 (11/59)			
High birth weight, 4.0+	6.9 (2/29)	6.8 (4/59)			
Sex of baby^a^ (%)					
Female	41.4 (12/29)	45.8 (27/59)	1.00	1.00	1.00
Male	58.6 (17/29)	54.2 (32/59)	1.195 (0.486–2.937)	0.834 (0.311–2.235)	0.956 (0.339–2.698)
Birth defects (%)	0 (0/29)	0 (0/59)	-	-	-

Values are shown as n (%). ^a^ Rough Adjusted for age and BMI. ^a^ Fine Adjusted for age, BMI, number of ET cycles, number of ET embryos, endometrium thickness and embryo stage.The reference variable within each sub-category has been arbitrarily designated and given the odds ratio of 1

### Effects of CsA on birth and infant characteristics

As for the birth and infant characteristics among successful pregnancy cycles (Table 2), the baby take-home rate [44.8 % vs. 38.3 %, *P* = 0.711] was not higher in the CsA group than in the control group [fine adjusted OR 0.872, 95 % CI 0.423–1.798]. Both groups were similar in terms of the singleton [80.8 % vs. 71.7 %] and multiple births rate [19.2 % vs. 28.3 %, p = 0.815, fine adjusted OR 0.840, 95 % CI 0.197–3.590]. There was no statistical difference in the gestational ages at birth [normal delivery rate:73.1 % vs. 80.4 %; preterm delivery rate:19.2 % vs. 19.6 %, *P* = 0.500, fine adjusted OR 1.668, 95 % CI 0.377–7.373], birth weight [normal weight: 72.4 % vs. 74.6; abnormal weight: 27.6 % vs. 25.4 %, *P* = 0.336, fine adjusted OR 1.834, 95 % CI 0.533–6.307] or sex of the baby [female: 41.4 % vs. 45.8 %; male:58.6 % vs. 54.2 %, *P* = 0.932, fine adjusted OR 0.956, 95 % CI 0.339–2.698] between pregnancies that occurred with and without CsA treatment. No birth defects were observed in this study.

### Multivariate logistic regression analysis

A binary logistic regression model was used to analyse embryo implantation and clinical pregnancy outcomes. Univariate logistic regression was used to identify variables related to outcomes and existing confounders. Moreover, variables with a univariate *P* value ≤ 0.10 and with known and potential confounders (Table 3) were included in the multivariate binary logistic regression model. CsA was not found to be an independent predictor for embryo implantation [fine adjusted OR 1.251, 95 %CI 0.739–2.120; *P* = 0.404] or clinical pregnancy [fine adjusted OR 1.634, 95% CI 0.772–3.458; *P* = 0.199] after adjusting for maternal age, BMI, number of ET cycles, number of embryos transferred, endometrium thickness, and embryo stage.

**Table 3:** Multivariate binary logistic regression analysis associations for patients with and without CsA

	Implantation^a^		Clinical Pregnancy^a^	
	Fine Adjusted OR (95 % CI)	*P* value	Fine Adjusted OR (95 % CI)	*P* value
CsA	1.251 (0.739–2.120)	0.404	1.634 (0.772–3.458)	0.199
Patients’ age at OPU (y)	0.579 (0.399–0.841)	0.004	0.411 (0.234–0.721)	0.002
Patients’ age during ET (y)	1.589 (1.097–2.303)	0.014	2.149 (1.233–3.745)	0.007
BMI (kg/m^2^)	1.078 (1.005–1.157)	0.036	1.069 (0.966–1.183)	0.195
No. of ET cycles	0.584 (0.344–0.993)	0.047	0.641 (0.315–1.307)	0.222
No. of embryos transferred	0.515 (0.243–1.092)	0.083	2.171 (0.924–5.099)	0.075
Endometrium thickness(mm)	0.976 (0.866-1.100)	0.690	0.910 (0.773–1.072)	0.261
Embryo stage	1.546 (0.945–2.530)	0.083	1.904 (0.937–3.867)	0.075

*CsA* Cyclosporin A; *OPU *oocyte pick-up; *ET* embryo transfer; *BMI* body mass index. ^a^ Fine Adjusted for age, BMI, number of ET cycles, number of ET embryos, endometrium thickness and embryo stage.Multivariate analysis included variables with univariate *P* value ≤ 0.10 and confounders those were known and potential

## Discussion

Maternal immune tolerance towards a semi-allograft embryo is a prerequisite for normal pregnancy, and an imbalance of the maternal-foetal immune interaction has been verified as an important cause of pregnancy-related diseases [1–6, 36]. Thus, CsA, a typical immunosuppressant, has been introduced to treat some types of pregnancy failures [5]. However, the indications, effectiveness, and safety of CsA in the treatment of transfer failure remain unclear.

Since this was a retrospective cohort study, we used binary logistic regression to explore the relationship between CsA treatment and pregnancy outcomes. It was found that the application of CsA did not significantly improve the implantation [46.7 % vs. 39.2 %, *P* = 0. 404] or clinical pregnancy rate [63.8 % vs. 47.5 %, *P* = 0.199] in patients. The patient’s age at oocyte pick-up and ET rather than CsA were two real independent predictors of implantation and clinical pregnancy rate. These results indicate that CsA has no beneficial effects on embryo implantation in patients with a history of unexplained transfer failure.

This conclusion was different from that of other studies and our previous findings[30, 37]. Our previous animal investigation showed the positive role of CsA in improving the biological functions trophoblast cells and inducing maternal-foetal tolerance in miscarriage-prone mice with a high mRNA level of CD80/CD86 [30]. Azizi et al.[37] reported that CsA improves pregnancy outcomes in a study of 76 RPL patients with raised Th1/Th2 ratios. In these studies that reported a beneficial effects of CsA on pregnancy outcomes in patients with recurrent pregnancy loss and excessive activation of the maternal immune system were observed. Therefore, the pregnancy-promoting effect of CsA in such studies could be attributed to the immunosuppressive effect that protected the embryos from an attack by the maternal immune system. However, the factors influencing embryo implantation are complex, and some are unknown. In this study, the included patients had no obvious immune-related disease and were classified as having unexplained transfer failure. Thus, we speculate that CsA may have different effects in different types of transfer failures and may only be effective in certain types of patients. In addition, one study that showed that implantation rates in a setting of FET are similar and do not change significantly between the first, second, and third transfers, suggesting that true recurrent implantation failure (RIF) is rare [38] ; thus, we cannot completely rule out that a lack in the difference of effect between the CsA and control groups could have been because patients with one prior failed transfer may not have any underlying unexplained implantation issue. Since we enrolled patients who had accepted at least one transfer with high-quality embryos but failed to conceive, our study does not provide insight into the possible benefit of CsA in a population with true RIF.

As for the effects of embryo quality on pregnancy outcomes, we recruited only those patients who underwent ET using advanced-stage embryos in this study. However, we did not perform invasive chromosome screening on these transferred embryos because of our limited research conditions, which, if performed, could have reduced the number of transfer failures and abortions caused by chromosomal abnormalities in the embryos. It is still unclear whether there is any difference between the chromosomes of the embryos of the two groups. Further, although CsA did not improve the clinical outcome in patients with high-quality ET, in light of its ability to promote trophoblast function [29, 31], whether it is beneficial in implantation of low-quality embryos with a relatively lower developmental competence needs further investigation.

Other issues that need attention are the side effects and toxicity of CsA. Fortunately, as CsA has been used with a long-term application in organ transplant patients, much attention has already been paid to the developmental toxicity and teratogenicity of this medicine [18, 39, 40]. The National Transplantation Pregnancy Registry (NTPR) reported that in female recipients, no specific pattern of malformation in their new-borns or no apparent increase in the incidence of small-for-gestational-age new-borns was observed. Thus, the NTPR suggested that if there is a stable graft function, additional successful pregnancies will be possible [40]. This study also analysed some birth and infant characteristics to explore the safety of CsA. We observed no difference in birth weight, sex ratio or preterm births between the CsA and control groups. No birth defects were observed in the this study. The duration of CsA treatment was much shorter in transfer failure patients than that in organ transplant patients. Therefore, short-term application of CsA might be safe in patients undergoing ET treatment. A mouse embryo experiment [39] revealed that CsA showed toxic effects on embryonic growth after the concentration reached 10 µg/mL. Therefore, it is recommended that we carefully choose the doses of CsA in women who accept ART. Although no obvious side effects were found in the new-born babies, offspring health is a serious issue, and we still need a long time to evaluate the potential benefits and hazards of using CsA in clinical practice.

The inherent limitations of this retrospective study include the unavoidable nature of patients’ selection of treatment and a small sample size, which might have led to selection bias. Although we had set strict inclusion and exclusion criteria to screen factors that could interfere with our results, we suggest that adequately powered multi-centre randomised clinical trials with long-term follow-up of offspring should be conducted to obtain more precise information about the application of CsA in ART patients.

## Conclusions

Our study indicates that CsA has no beneficial effects on the clinical pregnancy outcomes in patients with a history of unexplained transfer failure. Although CsA does not affect infant characteristics, its clinical application in assisted pregnancy therapy requires careful consideration.

## Data Availability

The datasets used and/or analysed in this study are available from the corresponding author on reasonable request.

## Abbreviations

ART: assisted reproductive treatment.
CsA: Cyclosporin A.
FET: frozen-thawed embryo transfer.
IVF: in vitro fertilization.
ICSI: introcytoplasmic sperm injection.
BMI: body mass index.
FSH: follicle stimulating hormone.
HCG: human chorionic gonadotropin.
